# Diseases of the Kidney

**Published:** 1901-11-02

**Authors:** 


### Diseases of the Kidney.

**Treatment.**—Gossner[1] relates a case of hæmaturia treated by gelatine injections. The urine was otherwise normal, and the blood came from one kidney only. After other remedies had failed, 200 c.c. of sterilised gelatine solution were injected into the thoracic wall, and the hæmorrhage quickly ceased entirely. It should be added that severe pains, vertigo, and restlessness followed the injection. T. R. Glynn[2] records a successful case of uræmic convulsions treated by bleeding and saline injections, a pint being injected subcutaneously, and a pint into the rectum, after 20 ounces of blood had been abstracted from the arm. The patient, who was the subject of acute nephritis, was soon passing 70 ounces of urine per diem, and made a complete recovery, getting rid of his albuminuria and high-tension pulse entirely. The free administration of oxygen is recommended where venesection is unadvisable. The abstractor has seen another instance where temporary recovery was obtained by the injection of large quantities of very hot water into the rectum, with the result that 320 ounces of urine were passed next day. Urotropine is more and more used both in ammoniacal decomposition of urine, in prostatitis with residual urine, in enteric to destroy the bacilli and prevent the spread of infection, and in cystitis, though it is of little value in pyonephritis, gonorrhœal cystitis, or tuberculosis. Bacilli coli and streptococci are less affected by it than other organisms.[3] Phosphaturia is often relieved by it. Messrs. Marie and Guillain[4] report a temporary cure of uræmic headache by lumbar puncture which relieved the high tension of the cerebro-spinal fluid. Another case was reported at the same meeting, and it seems that the operation might be employed in many uræmic conditions, and for headache due to some forms of cerebral tumour.

**Interstitial Nephritis.**—H. McLaren[5] records an instance of dysenteric ulceration of the large intestine such as has been occasionally seen in this disease. The mucous membrane was dark in colour, swollen, infiltrated with leucocytes, and showed small abscesses. L. Guthrie[6] has met with a ninth case of interstitial nephritis in a child aged seven. There were typical cardiac and renal symptoms, a bronzed skin and wasting, followed by right- and finally left-sided convulsions. Large cerebral hæmorrhages were found after death. He thought the disease in such cases might arise from acute interstitial nephritis, or more probably from syphilis. This child had suffered much from intestinal trouble as an infant, but her illness was only noticed three months before death. The vomiting, headache, and loss of sight were marked, and the heart was greatly hypertrophied. A practical point is that, if early syphilis is prone to affect the kidneys, care should be taken in giving mercury. Though the child may flourish on it for a few weeks, sudden death from uræmia may follow the action of the drug on the weakened kidneys. A. G. Barrs[7] holds that acute nephritis ends either in recovery or death, while chronic forms are chronic from the beginning, and that the quantity of albumen or urea is not of great clinical importance. He does not think that the cardio-vascular changes are so common as is generally believed, and they, together with cerebral hæmorrhage, may be produced in the absence of any kidney disease by quite different causes. Barrs allows ordinary full diet as long as the bowels act freely, and gives hot-air baths with the systematic use of purgatives and occasional venesection. A case of the *small white kidney* in a boy of 15 is discussed by W. H. Brazil,[8] who remarks on the combination of œdema, pallor, and albuminuria as seen in the large white; with hypertrophy of the heart and hard pulse seen in the small red one. The œdema gradually increased and the patient died from uræmic convulsions. The patient was known to have had scarlet fever ten years before, without, however, any subsequent illness till eight years later when he was found to have albuminuria. Post-mortem examination showed that the kidneys were nearly white, lobulated, and without any cysts. The capsule peeled easily, and both the cortex and the medulla were greatly diminished. He would regard this form of kidney disease as a late stage of the large white and not as a variety of the small red, from which it differs in so many respects. *Saturnine gout* is a state where two poisons act on the kidneys, and it is therefore not to be wondered at that Lorimer found albuminuria in 89 out of 107 cases, and in later stages it is, he thinks, always present. The effects of lead by itself on the kidney are well known and comprise both tubular and interstitial nephritis; indeed, Dickenson thinks that "no other cause of granular kidney is so efficacious." Gout alone is often accompanied by the same degeneration, which may sometimes be due to direct irritation by the gouty deposit; but when the two irritants act together the disease begins earlier and runs a more rapid course.

[1] Brit. Med. Jour., April 6. [2] Brit. Med. Jour., Jan. 26. [3] Therapist, March 15. [4] Lancet, May 25. [5] Glasgow Med. Jour., Feb. [6] Clinical Jour., March 6. [7] Brit. Med. Jour., March 30. [8] Med. Chron., June. [9] Quarterly Med. Jour., p. 245. [10] Lancet, Feb. 23. [11] New Zealand Med. Jour., Feb.

